# Thioredoxin and Cancer: A Role for Thioredoxin in all States of Tumor Oxygenation

**DOI:** 10.3390/cancers2020209

**Published:** 2010-03-25

**Authors:** Therese Christina Karlenius, Kathryn Fay Tonissen

**Affiliations:** 1 School of Biomolecular and Physical Sciences, Griffith University, Nathan, QLD, 4111, Australia; E-Mail: therese.karlenius@student.gu.edu.au (T.K.); 2 Eskitis Institute for Cell and Molecular Therapies, Griffith University, Brisbane Innovation Park, Don Young road, Nathan, QLD 4111, Australia

**Keywords:** thioredoxin, tumor, hypoxia, oxidative stress, preconditioning, cycling hypoxia

## Abstract

Thioredoxin is a small redox-regulating protein, which plays crucial roles in maintaining cellular redox homeostasis and cell survival and is highly expressed in many cancers. The tumor environment is usually under either oxidative or hypoxic stress and both stresses are known up-regulators of thioredoxin expression. These environments exist in tumors because their abnormal vascular networks result in an unstable oxygen delivery. Therefore, the oxygenation patterns in human tumors are complex, leading to hypoxia/re-oxygenation cycling. During carcinogenesis, tumor cells often become more resistant to hypoxia or oxidative stress-induced cell death and most studies on tumor oxygenation have focused on these two tumor environments. However, recent investigations suggest that the hypoxic cycling occurring within tumors plays a larger role in the contribution to tumor cell survival than either oxidative stress or hypoxia alone. Thioredoxin is known to have important roles in both these cellular responses and several studies implicate thioredoxin as a contributor to cancer progression. However, only a few studies exist that investigate the regulation of thioredoxin in the hypoxic and cycling hypoxic response in cancers. This review focuses on the role of thioredoxin in the various states of tumor oxygenation.

## 1. Introduction

Both oxidative stress and hypoxic microenvironments are commonly found in tumors. These regions are often associated with elevated levels of antioxidants, especially members of the Thioredoxin (Trx) system, and accumulating evidence indicates that the Trx system plays an important role in tumor progression and metastasis. This review will focus on the involvement and regulation of the Trx system in the different oxygenation states of tumors.

## 2. Oxygen Homeostasis

Oxygen homeostasis is essential for the survival of all aerobic organisms. However, this balance in a cell can be disrupted by either an increase or decrease in the level of oxygen. Therefore, adaption to the environmental oxygen availability is of crucial importance in controlling cellular homeostasis. Cells utilize distinct mechanisms to adapt to either an increase or a decrease in cellular oxygen levels.

Aerobic organisms continually metabolize oxygen through several oxidative systems, for example the NADPH oxidases [1], the xanthine/xanthine oxidase system [2] and the respiratory chain within the mitochondria [3]. In many cases, however, oxygen undergoes incomplete one-electron reduction to form a number of highly reactive molecules commonly called Reactive Oxygen Species (ROS). ROS includes free radicals with unpaired electrons, such as the superoxide anion, hydroxyl radical and oxidants such as hydrogen peroxide (H_2_O_2_), all of which are inherently unstable and often highly reactive [4]. Even under normal physiological conditions ROS molecules are produced within cells.

ROS have some beneficial roles through involvement in intracellular signaling and redox regulation of the cell. For example, H_2_O_2_ and the superoxide anion are redox regulators of transcription factor activities, and several cytokines, growth factors, hormones and neurotransmitters employ ROS as secondary messengers in intracellular signal transduction [5,6,7]. On the other hand, ROS can also cause significant damage within the cell, such as damage to DNA, oxidation of lipids and oxidation of amino acids in proteins [8]. To defend themselves, cells utilize several distinct antioxidant systems. Antioxidants are molecules that counteract excessive ROS production by preventing or reducing the oxidation of ROS targets. Therefore, in normal physiological conditions, the cellular oxidation-reduction (redox) equilibrium in aerobic cells is maintained by ROS and antioxidants [9,10]. However, under certain conditions ROS levels can increase dramatically and as a consequence so-called oxidative stress occurs. 

### 2.1. Oxidative Stress Response

**Figure 1:**
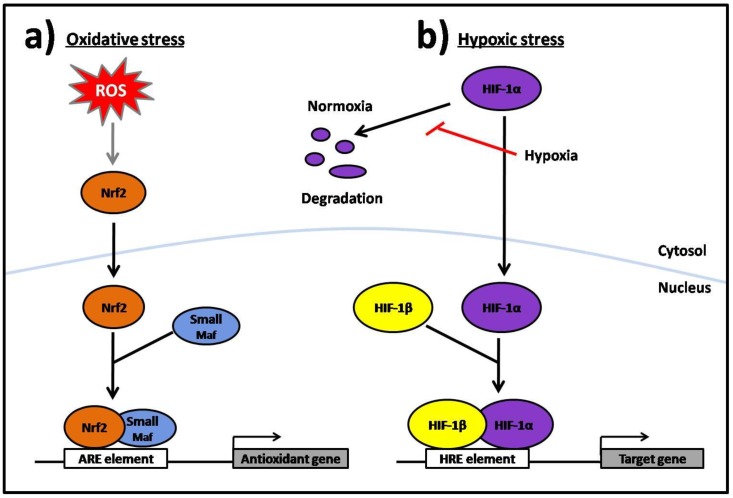
(a) Schematic presentation of the Nrf-2/ARE pathway. In response to oxidative stress ROS causes nuclear translocation of Nrf-2, which then forms a heterodimer with a small Maf protein. This complex then interacts with the ARE element and induces transcription of its target antioxidant genes. **(b)** Schematic presentation of the HIF-1/HRE pathway. Under normoxia HIF-1α is rapidly degraded in the cytosol. In response to hypoxia HIF-1α becomes stabilized, translocates to the nucleus where it dimerizes with HIF-1β and then binds to the HRE element and induces transcription of its target genes.

In times of oxidative stress cells respond by inducing the expression of several antioxidant systems in order to re-establish the intracellular redox homeostasis [8,9]. The regulatory element mainly responsible for this simultaneous induction is the antioxidant responsive element (ARE), which is usually found in the promoter region of the induced target genes [11]. The transcription factor that regulates the induction of these genes *via* the ARE element is Nrf-2 (NF-E2 related factor). Upon oxidative stress, Nrf-2 forms heterodimers with small Maf proteins in the nucleus and then binds with ARE-containing gene promoters to induce their expression [12,13,14] (Figure 1a). Consequently, many antioxidant systems, including the thioredoxin system, are simultaneously up-regulated *via* the Nrf-2/ARE pathway in response to oxidative stress. 

### 2.2. Hypoxic Stress Response

Disruption of the redox homeostasis within cells can also occur when oxygen demand exceeds supply, a condition termed hypoxia [15]. The cells defense mechanism against this unfavorable condition differs from that of oxidative stress. Instead of activating the Nrf2/ARE pathway the adaption to hypoxic stress is primarily mediated through the hypoxia-inducible factors (HIFs). These HIFs bind to the hypoxia-response element (HRE) sequences in the promoter or enhancers of target genes and up-regulate their expression. HIF-1 is composed of two subunits. The HIF-1β subunit is constitutively expressed in the cell while the HIF-1α subunit is regulated in an oxygen-dependent manner [16,17] (Figure 1b). The main target genes up-regulated in response to hypoxic stress *via* the HIF-1/HRE pathway are involved in anaerobic metabolism, angiogenesis and haematopoiesis. Importantly, all these target genes ensure that either the cell restores oxygen homeostasis by surviving with minimal energy production, or that the cells die due to persistent lack of energy [15]. Interestingly, the thioredoxin system also seems to play important roles in the hypoxic response (see thioredoxin and hypoxia). 

## 3. The Thioredoxin System

The thioredoxin system is comprised of the redox-active protein thioredoxin (Trx), the enzyme thioredoxin reductase (TrxR) and NADPH. This antioxidant system is essential for normal cellular functions which is evident by the observation that Trx knockout mice are embryonic lethal [18]. The Trx system plays an important role in many cellular functions, including redox control of transcription factors, synthesis of deoxyribonucleotides, cell growth and protection against oxidative stress [10,19,20,21].

**Figure 2:**
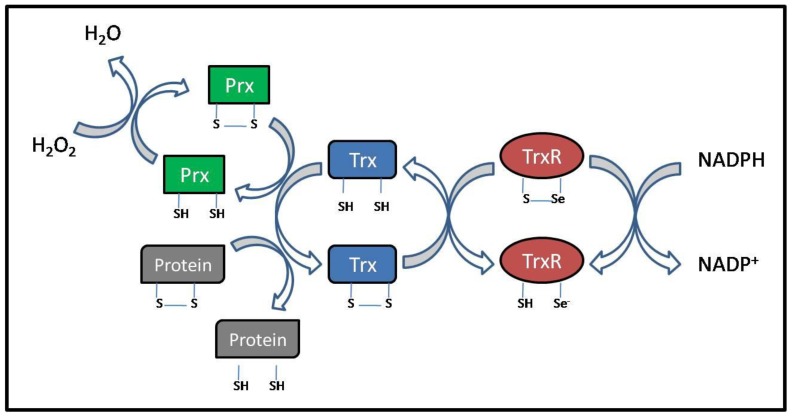
Mechanism of action of the thioredoxin (Trx) redox system. Reduced Trx catalyzes the reduction of disulfides (s-s) within oxidized cellular proteins, such as peroxiredoxin (Prx). In this process Trx becomes oxidized which in return is reduced by thioredoxin reductase (TrxR) at the expense of NADPH.

Thioredoxin is a small (~12 kDa) ubiquitously expressed protein which is conserved through all species, from Archebacteria to humans [19]. All Trxs have a redox active site of Cys-Gly-Pro-Cys, which can reduce disulfides in proteins and peptides [22,23]. Reduced Trx catalyses the reduction of ROS oxidized cysteines of many proteins both intracellularly and extracellularly and in this process Trx itself becomes oxidized. Trx can then be reduced by the action of TrxR at the expense of NADPH [19,24] (Figure 2). To date TrxR is the sole known electron donor for Trx.

Three isoforms of Trx have been identified in mammalian cells, with all containing the conserved active site. They are Trx1, a cytoplasmic protein [25]; Trx2, a mitochondrial protein [26]; and SpTrx, which is expressed mainly in spermatozoa [27]. Mammalian cells also contain three isoforms of TrxRs. They are the cytosolic protein TrxR1, the mitochondrial protein TrxR2 and the testis specific thioredoxin glutathione reductase (TGR) [28]. Mammalian TrxRs can reduce a number of small-molecule substrates in addition to their role in maintaining Trxs in their reduced forms [10]. These substrates include ascorbic acid, lipid hydroperoxides, α-lipoic acid, and hydrogen peroxide. The thioredoxin-1 system consists of Trx1 and TrxR1. Unless otherwise indicated, Trx and TrxR refers to the cytosolic Trx1 and TrxR1 in this review.

Thioredoxin can exert its antioxidant function through either directly quenching singlet oxygen and scavenging hydroxyl radicals, or indirectly by reducing oxidized ROS target proteins [23,29]. However, Trx performs most of its antioxidant functions through peroxiredoxins (Prx), also called thioredoxin peroxidases [30,31,32]. Prx uses the SH groups as reducing equivalents and aid in the direct reduction of peroxides, such as H_2_O_2_ and different alkyl hydroperoxides. The oxidized form of Prx can then be recycled back to its reduced form by Trx [33] (Figure 2).

Trx can exist in the extracellular environment, cytoplasm and nucleus. However, Trx has distinct roles in each of the different environments [24]. Extracellular Trx exhibits chemokine like activity [34], while in the cytoplasm Trx regulates the redox balance of the cell and also the activity of certain proteins [35,36]. In the nucleus, Trx has been shown to interact with many transcription factors and thereby regulate gene expression [36]. Hence, Trx is responsible for the maintenance of many important cellular processes that are dependent on thiol-redox states. 

## 4. Thioredoxin and Transcription Factors

Transcription factors are proteins that regulate cellular functions by altering the gene expression profile. Thereby cells can modulate their transcriptome to adjust to normal physiological and pathophysiological changes in oxygen levels. Several transcription factors are activated by Trx through redox regulation, which modulates their DNA binding activities. 

Thioredoxin can directly reduce some transcription factors, while other transcription factors use Ref-1 (redox factor-1) as an intermediate. A specific cysteine residue(s) in these transcription factors is reduced by Ref-1, which results in enhanced DNA-binding activity. In order for Ref-1 to catalyze this reduction it needs to be in its reduced form, which is catalyzed by Trx [37,38,39]. Transcription factors dependent on the Trx/Ref-1 interaction are responsible for the activation of many genes that have the overall effect of promoting cell viability in response to adverse conditions including oxidative stress and hypoxia. In addition to its function as a major redox-signaling factor, Ref-1 is also a DNA-repair endonuclease. It is involved in the base excision repair (BER) pathway [37]. The BER pathway is responsible for repair of apurinic/apyrimidinic (AP) sites in DNA, which are a major end product of ROS damage. Therefore, Trx has several important functions in protecting the cells from oxidative stress *via* Ref-1. 

**Figure 3:**
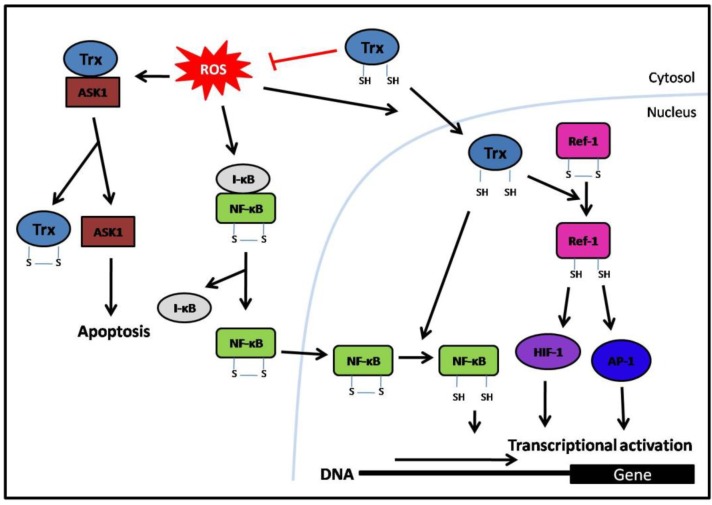
Thioredoxin (Trx) and redox signaling of transcription factors. Trx negatively regulates apoptosis in the cytoplasm *via* redox regulation of ASK-1 and inhibition of Iκβ degradation by scavenging ROS. Under oxidative conditions, Trx is translocated to the nucleus where it increases the DNA binding activity of NF-κβ *via* reduction of its cysteine residue. Trx also increases the DNA binding activity of other transcription factors, such as AP-1 and HIF-1, indirectly *via* the intermediate Ref-1. Red lines indicate an inhibitory effect. S-S = oxidized form. SH = reduced form.

One of the transcription factors that is dependent on the Trx/Ref-1 interaction is Activator protein-1 (AP-1) [40]. AP-1 is not a single transcription factor but rather is comprised of various homo- or heterodimers formed between the proteins of the basic region-leucine zipper (bZIP) family. The dimeric complexes are predominantly composed of Jun homodimers and Jun-Fos heterodimers [41,42]. Since both Jun and Fos families contain multiple members, the AP-1 transcription factor is involved in a wide range of physiological functions. For example, AP-1 regulates the expression of genes involved in cell growth in response to external stimuli [43]. The DNA binding of AP-1 is regulated by the redox state of a cysteine residue within the DNA binding domain of both proteins making up the dimer. Trx reduces these cysteines indirectly *via* Ref-1 and thereby increases the DNA binding activity of AP-1 [40,44] (Figure 3). Thus, Trx also contributes to cell growth.

Another important transcription factor regulated by Trx through Ref-1 is HIF-1α, which controls the expression of hypoxic stress-responsive genes. Redox modification of a single cysteine residue within the HIF-1α subunit of HIF-1 is necessary for the direct interaction of HIF-1 with the CBP/p300 co-activator that leads to increased expression of its target genes *via* the hypoxic response element (HRE) [45]. Trx, through Ref-1, is indirectly responsible for this modification (Figure 3).

Trx can also act directly on some transcription factors without the requirement for Ref-1. For example, Trx can influence apoptosis by activating nuclear factor (NF)-κB, which regulates the expression of genes that antagonize cell death [46,47,48]. In the cytoplasm, ROS mediates degradation of the inhibitor IκB to activate NF-κB nuclear translocation. Since Trx is an antioxidant it scavenges ROS in the cytoplasm, hence inhibiting the degradation of IκB. However, in response to NF-κB activation stimuli Trx translocates from the cytoplasm to the nucleus where instead it activates NF-κB DNA binding (Figure 3). Trx directly reduces a cysteine of NF-κB and allows NF-κB-dependent gene expression [36,49]. Furthermore, Trx interacts directly with the apoptotic pathway through binding to ASK-1 (apoptosis signal-regulating kinase-1), a member of the MAPKKK family [50]. Reduced Trx physically interacts with ASK-1 in a redox-dependent manner and inhibits its activity. When Trx is oxidized by ROS it dissociates from ASK-1, which is then activated to transduce the apoptotic signal (Figure 3). This ROS-induced apoptosis is a defense mechanism against persistent oxidative stress. 

## 5. Thioredoxin and Oxidative Stress

In times of oxidative stress Trx, to some extent, reduces intracellular proteins and lowers levels of ROS as part of the antioxidant defense. Trx also translocates to the nucleus in response to oxidative stress where it can exert regulatory functions of transcription factors *via* redox modifications [36,51,52]. By regulating the activity of several transcription factors Trx can influence many important cellular functions in response to oxidative stress, including DNA repair, cell growth and proliferation, and apoptosis [53].

It is well established that the gene expression of Trx is induced by oxidative stress and that this induction is mediated mainly by the binding of Nrf2 in the ARE element present in the Trx promoter [54,55]. The TrxR and Prx promoters also contain ARE elements, which are responsible for their induced expression in response to oxidative stress [32,56,57,58]. It is worth noting that Trx in a reduced state can enhance the binding of Nrf2 with the ARE, thus activating it [59]. Furthermore, Ref-1 expression is up-regulated in response to oxidative stress [60,61]. Therefore oxidative stress leads to increased levels of Trx, which in turn activates the transcription factors responsible for inducing even higher levels of Trx and other antioxidants.

It is also well known that cancer cells in general are under increased oxidative stress compared to normal cells. Increased oxidative damage of DNA bases has been found in many cancerous tissues [62,63]. Additionally, *in vitro* human tumor cell lines have been found to produce significantly greater amounts of ROS than non-transformed cell lines, thereby keeping these cells under persistent oxidative stress [64]. These higher levels of ROS in cancers are usually associated with extremely high levels of Trx and other redox control proteins, which will be discussed later (See Thioredoxin and cancer).

## 6. Thioredoxin and Hypoxia

Hypoxia occurs when the levels of oxygen in a cell falls below the normal levels of oxygen tension. Although hypoxia is generally harmful to the cell by disrupting the cells redox homeostasis, it does have some beneficial roles as well. For example, hypoxia is encountered in embryogenesis, in which hypoxia signaling is considered to be necessary for normal development [65]. 

In response to hypoxia, the HIF-1 protein is stabilized and induces the expression of genes that function to improve oxygenation through angiogenesis and erythropoiesis. The availability of HIF-1 is mainly determined by the HIF-1α subunit, which is usually suppressed due to its rapid, oxygen-dependent degradation, while the HIF-1β subunit is not controlled by oxygen levels. During hypoxia, the HIF-1α protein stabilizes, translocates to the nucleus, dimerizes with HIF-1β and binds to the target DNA sequence within the promoter or enhancer region of different genes [66,67,68,69] (Figure 1b).

**Figure 4:**
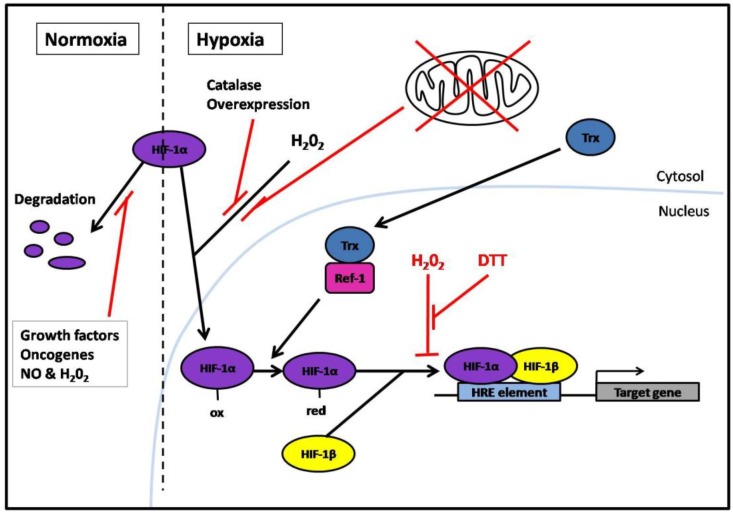
Thioredoxin (Trx) and ROS in the hypoxic stress response. Under normoxia HIF-1α is degraded in the cytosol while in hypoxia HIF-1α becomes stabilized and translocates to the nucleus. In hypoxia, the presence of H_2_O_2_ in the cytosol is necessary for HIF-1α stabilization. In the nucleus, reducing conditions are necessary for HIF-1 DNA binding. Trx reduces Ref-1 in the nucleus, which in turn reduces the HIF-1α subunit, which then dimerizes with HIF-1β to form HIF-1. The HIF-1 protein binds to the HRE element and induces transcription of its target genes. Red lines indicate an inhibitory effect. Red cross indicates non-functional mitochondria.

The HIF-1 protein is also regulated by many other factors aside from hypoxia, including oncogenes, growth factors, and quite paradoxically by free radicals such as the superoxide anion, H_2_O_2_ and NO. For example, exposure to NO has been shown to nitrosylate a specific cysteine residue in the HIF-1α subunit under normoxic conditions [70], thereby inhibiting its degradation. Furthermore, administration of H_2_O_2_ in human 293 cells caused HIF-1α stabilization and expression of HRE-luciferase under normoxic conditions [71]. Hence, ROS can stabilize HIF-1α under normoxic conditions (Figure 4). 

Increasing evidence demonstrate that ROS formation by the mitochondria is also involved in HIF-1α stabilization during hypoxia [72,73], although the increase in ROS seems to be to a much lesser extent than that occurring in response to oxidative stress [74]. One study showed that cells containing non-functional mitochondria cannot increase HIF-1α levels in response to hypoxia [71]. Another study using RNAi to suppress expression of the Rieske iron-sulfur protein of the mitochondrial complex III showed that ROS formation and HIF-1α expression levels decreased when exposed to hypoxia. However, addition of H_2_O_2_ to the cells resulted in an increase in HIF-1α protein under hypoxia [75]. Also, H_2_O_2_ inhibition during hypoxia in human 293 cells, through catalase over-expression, attenuated HRE-luciferase expression [71] (Figure 4). These observations suggest that the presence of H_2_O_2_ in the cytosol is necessary for HIF-1α stabilization during hypoxia. 

In contrast, several studies have shown that HIF-1 DNA binding requires reducing conditions in the nucleus. For example, treatment of purified HIF-1 with H_2_O_2_ or diamide abolished HIF-1 DNA binding activity. However, prior addition of dithioretiol (DTT) circumvented this effect [76] (Figure 4). These results suggest that HIF-1 itself is redox-sensitive. Indeed, as mentioned previously, the antioxidant proteins Trx and Ref-1 have been shown to enhance the transcriptional activation of HIF-1 during hypoxia through the modulation of a single cysteine residue in the HIF-1α subunit (Figure 4). Furthermore, a mutation of this Ref-1 target cysteine residue prevented the decrease in HIF-1α activity in response to hydroxyl radicals [77]. These results indicate that Trx plays an important role in the hypoxic-stress response.

In addition, evidence that Trx influences HIF-1 during hypoxia has been shown in several studies using cells over-expressing Trx. These cells showed increased HIF-1α levels, enhanced HIF-1 DNA binding and increased activation of HIF-1 target genes, such as vascular endothelial growth factor (VEGF) [78] and cyclooxygenase-2 (COX-2) [79]. In contrast, studies using a redox-inactive Trx clearly showed decreased HIF-1α protein levels [78]. Additionally, Trx and TrxR inhibitors have been shown to down-regulate expression of HIF-1α and its subsequent activity [80], which further emphasizes Trx’s role in HIF-1α regulation. 

These results are also consistent with other studies demonstrating that Trx and related redox proteins are up-regulated in response to hypoxia. For example, when HT-29 cells were exposed to hypoxia both Trx and TrxR mRNA expression were increased by 14-fold and 4-fold, respectively [81]. In lung cancer cells both mRNA and protein levels of Trx and Prx were up-regulated following hypoxia [82]. Up-regulation of Trx and Ref-1 has also been observed in hypoxic microregions of tumors from cervical cancer biopsy specimens [83]. However, the mechanism for the hypoxic induction of Trx is not fully understood. There is no evidence indicating that this induction is regulated by HIF-1. 

In summary, these results represent an important link between hypoxic and redox control processes and suggests that the roles ROS and Trx play in regulating HIF-1 during hypoxia are at least as important as the regulation ascribed to classical hypoxic induction pathways. 

## 7. Thioredoxin and Cancer

It is widely accepted that both oxidative stress and hypoxia are common features of tumors. These conditions can partly be explained by a growing tumor mass that quickly outgrows its vascular networks and therefore lacks oxygen and nutrients [84]. This decreased level of oxygen leads to the stabilization and activation of HIF-1, which in turn induces processes such as angiogenesis. Although angiogenesis occurs in nearly all human solid tumors, it does not occur in an efficient manner, leading to spatial and temporal inadequacies in delivery of oxygen [84]. Therefore, some regions of the tumor may contain chronic hypoxia, while other regions of the tumor may undergo cycling hypoxia, by switching between hypoxia and re-oxygenation conditions due to irregular flow of oxygen. The re-oxygenation phase following hypoxia inadvertently causes oxidative stress. 

Since cancer cells are often under high oxidative or hypoxic stress it is not surprising that they also express high levels of antioxidant proteins, including Trx, Prx and Ref-1. For example, Trx expression is increased in several primary cancers, including lung [82], cervix [83], pancreatic [85], colorectal [86], hepatocellular carcinomas [87], gastric carcinomas [88] and breast cancer [89]. The up-regulation of Trx and related proteins has been postulated to present a dynamic redox change to benefit proliferation and malignant progression of tumors.

Several studies implicate over-expression of Trx as one of the enhancers of cancer cell growth, either through the direct stimulation of cancer cell growth or through the inhibition of cancer cell apoptosis. In one of these studies, MCF-7 breast cancer cells were transfected with a redox-inactive Trx construct. The Trx protein produced from this construct acts in a dominant-negative manner. Inoculation of the transfected MCF-7 cells into immunodeficient mice resulted in an almost complete inhibition of tumor formation and a reversal of the transformed phenotype of the cancer cells was evident [90]. Another study showed that the growth of MCF-7 cells is inhibited when treated with Arsenic trioxide (ATO), in a dose-dependent manner [91]. This decrease in MCF-7 cell growth was correlated with inactivation of TrxR, resulting in Trx oxidation and subsequently inactivation of the whole Trx system. In addition, a more recent *in vivo* study further highlights the importance of Trx in promoting cancer cell growth. When two human lung carcinoma cell lines, expressing either high or low Trx levels, were injected subcutaneously into SCID mice the extent of tumor growth correlated with the levels of Trx expressed by the injected cells [92]. The cells expressing low levels of Trx gave rise to smaller tumors while the cells expressing high levels of Trx gave rise to much larger tumors. Overall, these studies suggest that Trx has an active functional role in promoting cancer cell growth and that its increased expression is not just a consequence of cancer progression. 

High levels of Trx expression have also been correlated with highly invasive and metastatic tumor activity both *in vitro* and *in vivo* [92,93,94,95]. An *in vitro* study using a neuroblastoma cell line revealed a possible mechanism by which Trx can enhance the metastasis of cancer cells. Trx was shown to stimulate cell invasion in these cells and to promote overall matrix metalloproteinase (MMP) activity by preferentially inhibiting the MMP inhibitors [93]. An *in vivo* study using mice injected with two human carcinoma cell lines expressing either high or low levels of Trx further implicates Trx as an enhancer of tumors metastasis [92]. Tumor metastases were evident in the lung of mice injected with the higher Trx expressing cell line while no metastases were visible in mice injected with the lower Trx expressing cell line. In addition, another study performed on two prostate carcinoma cell lines showed that they possessed different redox phenotypes, with the more invasive cell line displaying a more reduced state [95]. Similarly, expression studies have also shown the highest levels of Trx expression in the most aggressive tumors isolated from patients diagnosed with either breast, melanoma, thyroid, prostate or colorectal cancer [94].

Furthermore, high levels of Trx and other antioxidant proteins are also correlated with cells displaying resistance to various chemotherapeutic agents, including doxorubicin, cisplatin, docetaxel and tamoxifen. For example, in one study, tumor tissues taken from breast cancer patients showed no significant correlation between the expression of p53, BRCA-1, or Bcl-2 and a response to docetaxel. However, tumors with high Trx expression showed a significantly lower response rate to docetaxel than those with low Trx expression [96]. In another study, gene expression profiling of 44 breast tumor samples treated with docetaxel was performed, with a total of 2453 genes analyzed. In this study the docetaxel resistant cells, nearly half of the samples examined, were characterized by elevated expression of redox genes, specifically glutathione S-transferase, Prxs and Trx [97]. 

Over-expression of Trx also results in patients developing resistance to cisplatin [98], by scavenging intracellular toxic oxidants generated by this anticancer agent. Furthermore, resistance of ovarian cancer cell lines, as well as gastric and colon cancer cells, has been associated with increased intracellular Trx levels [99,100]. These results suggest that Trx not only has an active role in cancer growth but also in cancer progression, through inhibition of apoptosis, stimulation of metastatic and invasive activity and through the involvement of chemotherapy resistance in cancer cells. Another important observation is that the aggressiveness of many tumors can be correlated with their redox phenotypes, which is characterized by the degree of Trx expression. Given the large number of roles for Trx in cancer cells, it is therefore not surprising that Trx, and other members of the Trx system, have been put forward as key targets for compounds designed to inhibit cancer growth, progression and metastasis.

While Trx itself has been regarded as a potential target [101,102], TrxR has been the focus of most Trx system inhibitors. An inhibition of TrxR leads to oxidation of Trx and thus altered functionality of the entire Trx system. A number of TrxR inhibitors have been tested in clinical trials with some approved by the FDA for use as cancer therapeutic reagents (as recently reviewed in detail [103,104,105]). Arsenic trioxide (ATO) is a compound approved by the FDA and has been used successfully to treat acute promyelocytic leukaemia [106]. ATO binds to and irreversibly inactivates TrxR, which correlates with an inhibition of the growth of MCF-7 breast cancer cells [91]. Gold compounds have been investigated for some time as cancer treatment options and a number, including auranofin, have TrxR as their preferred target [107]. Motexafin gadolinium is a porphyrin like molecule currently in clinical trials for treating various types of cancers and was also shown to inhibit TrxR [108]. A common feature of all of these potent TrxR inhibitors is that they primarily function as pro-oxidants and can cause serious side effects due to their high toxicity. An alternative approach for the future may lie in the emergence of natural products, including antioxidant rich food and beverages, which can be tolerated at much higher concentrations by the human body. Curcumin is one such example of a commonly used spice that exhibits anticancer effects and irreversibly inhibits TrxR function [109]. Recently, both green [110] and black teas [111] were found to contain antioxidant components that can inhibit TrxR and are capable of inhibiting HeLa cell growth *in vitro*. 

However, more information is needed regarding how these agents function against different types of cancers. The redox phenotype of different cancers is likely to be a major determinant of these agents’ selectivity to target particular cancers. Since the redox phenotype of cancers are characterized by the degree of Trx expression, which in turn is influenced by the oxygenation state of the tumor, the cycling occurring between hypoxia and oxidative stress may also be a major contributor to the cancers’ progression and its sensitivity to different cancer therapeutic reagents. 

## 8. Thioredoxin and Cycling Hypoxia

The oxygenation state of a tissue is a result of the balance between delivery and consumption of oxygen. Since angiogenesis in tumors is often abnormal this balance is regularly disrupted. Instead, tumors often have sparse arteriolar supply [112], inefficient orientation of microvessels [113], low vascular density, extreme variations in microvessel red blood cell flux [114] and increased blood viscosity. All of these factors lead to a highly unstable oxygen supply in tumors. Therefore, the most important feature of a tumor is that its oxygen supply is cyclical, a phenomenon often referred to as cycling hypoxia or intermittent hypoxia.

Cycling hypoxia was discovered almost 30 years ago and since then strong evidence has emerged demonstrating that the kinetics of cycling hypoxia are complex and have at least two dominant timescales [115,116,117], which superimpose on each other. One has a frequency of a few cycles per hour while the second timescale varies from hours to days. Recent studies have shown that fluctuations in red blood cell flux is primarily responsible for the faster frequencies [118], while vascular remodeling is responsible for the slower frequencies [119]. 

Most studies on cycling hypoxia have focused on the regulation of HIF-1 and angiogenesis. Several of these studies have shown an up-regulation of HIF-1 activity to a level that supersedes that of chronic hypoxia [120,121]. Since cycling hypoxia involves several re-oxygenation phases, it is tempting to speculate that increased levels of ROS and antioxidants would also occur. However, only limited studies have been performed with regards to the association of antioxidants with the dynamic changes of tumor oxygenation. 

### 8.1. Ischemic Preconditioning

**Figure 5:**
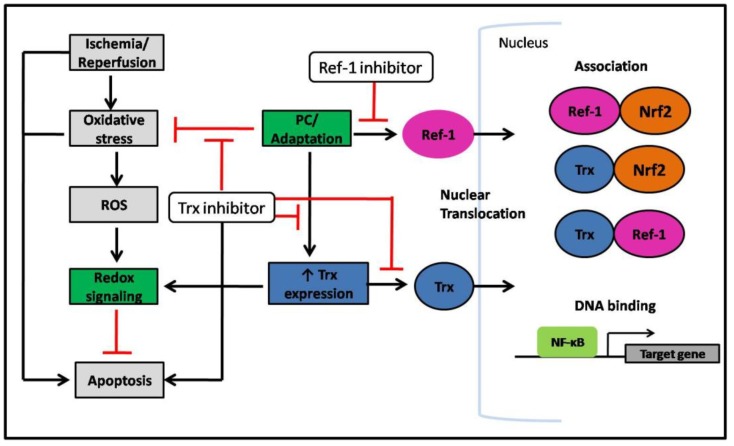
Schematic representation of the ischemic preconditioning survival signal mediated *via* Trx and Ref-1 redox signaling. Red lines indicate an inhibitory effect.

Most of the available data regarding antioxidants and cycling hypoxia come from studies of ischemia/reperfusion in cardiac disease [122,123,124,125,126]. Hearts exposed to cycles of short periods of ischemia followed by reperfusion become resistant to subsequent lethal ischemic injury. This technique is commonly used for myocardial preservation and is known as ischemic preconditioning (PC) [127,128,129,130]. The cardio-protective abilities of ischemic preconditioning have been shown to be redox regulated [131,132,133,134]. For example, ischemic preconditioning treated rat hearts showed an abolished survival signal when treated with the Trx inhibitor CDDP (an antitumor agent) and a significant number of apoptotic cardiomyocytes appeared in the preconditioned myocardium [135,136]. In addition, oxidative stress was significantly reduced in ischemic preconditioning treated hearts, while inhibition of Trx with CDDP increased oxidative stress in the hearts due to increased amount of malonaldehyde (Figure 5). Interestingly, myocardial adaption to ischemic stress was associated with an over-expression of Trx. 

Although there are several studies implicating a role of Trx in ischemic preconditioning survival signaling, its mechanism of action still needs to be elucidated. Interestingly, recent studies show that both Trx and Ref-1 are rapidly translocated to the nucleus in response to ischemic preconditioning treated rat hearts [137,138]. Injection of a small hairpin RNA against Trx abolished Trx nuclear translocation and antisense Ref-1 inhibited Ref-1 nuclear translocation [137]. In addition, association of Trx and Ref-1 occurred in the nucleus in response to ischemic preconditioning [138]. This study also showed increased DNA binding of NF-κβ and phosphorylation of Akt, which increased cell survival and these events were completely abolished when inhibiting either Trx or Ref-1. Furthermore, ischemic preconditioning treated hearts also showed a significant association in the nucleus between Ref-1 and Nrf2, which was decreased when treated with antisense Ref-1. Similarly, Trx and Nrf-2 association in the nucleus was observed, which was also decreased in the presence of antisense Ref-1 (Figure 5) [137]. 

In summary, these results indicate that cell survival signaling by cycles of ischemia/reperfusion in rat hearts is redox dependent. This survival signal seems to be mediated by ROS, which uses Trx and Ref-1 to transmit the signal. 

### 8.2. Hypoxic Preconditioning

There are a number of types of preconditioning that are being studied in different organs [139,140,141]. Some of these studies use ischemic preconditioning while others use hypoxic preconditioning. Hypoxic preconditioning refers to a short period of hypoxia followed by a period of re-oxygenation which leads to protection from a subsequent lethal hypoxic insult about 24–48 hours later [142]. Hypoxia is defined as a decrease in tissue oxygen concentration below normal, while ischemia is defined as a decrease in blood flow to a tissue that prevents adequate delivery of oxygen. Consequently the hypoxic and ischemic cellular responses differ from each other in that ischemic/reperfusion causes cardiac arrhythmias and systemic hypotension in animals while hypoxic/re-oxygenation does not [142]. Therefore the mechanisms of ischemic and hypoxic preconditioning differ to some extent, although both types appear to require synthesis of specific RNA and proteins [143,144,145,146,147]. Interestingly, many of the molecules implicated in the two types of preconditioning are also induced in response to hypoxia, for example HIF-1 and VEGF [148,149,150]. Since the mechanisms of ischemic/hypoxic preconditioning and cycling hypoxia in tumors are very similar one could therefore speculate that cycling hypoxia is a major contributor to the generation of resistant tumor cells to hypoxic-induced cell death. Additionally, adaption of tumors to cycling hypoxia may also promote tolerance to ROS generation during re-oxygenation periods. The tumor cell survival signal could potentially be mediated *via* the redox signaling of Trx/Ref-1 similar to that occurring in ischemic preconditioning treated rat hearts.

## 9. Conclusions

Thioredoxin protein levels are elevated in many human primary cancers and this high expression is associated with aggressive tumor growth and inhibited apoptosis, as well as decreased patient survival and resistance to anti-cancer treatments. Tumors can contain regions with either chronic hypoxia or oxidative stress due to abnormal vascular networks, and the fact that Trx expression is induced in response to both of these conditions further emphasizes the important role of Trx within cancers. It is widely accepted that Trx plays an important protective role in response to oxidative stress. In contrast, not many studies have been performed on the mechanisms of regulation of Trx in response to hypoxia or cycling hypoxia. Although cycling hypoxia in tumors has been suggested to have different biological consequences than hypoxia alone, it has for the most part been overlooked in many studies. Instead the vast majority of work relating to changes in oxygenation in tumors has been focused on the effect of hypoxia. Therefore, there is little known about the contribution of re-oxygenation to tumor progression, or the involvement of antioxidants in this process. Further investigations are needed to understand the complete role of Trx in the response to the dynamic changes of tumor oxygenation.

## References

[1] Nauseef W.M. (1999). The NADPH-dependent oxidase of phagocytes. Proc. Assoc. Am. Physicians.

[2] Kuppusamy P., Zweier J.L. (1989). Characterization of free radical generation by xanthine oxidase. Evidence for hydroxyl radical generation. J. Biol. Chem..

[3] Cadenas E., Davies K.J. (2000). Mitochondrial free radical generation, oxidative stress, and aging. Free Radic. Biol. Med..

[4] Fridovich I. (1999). Fundamental aspects of reactive oxygen species, or what's the matter with oxygen?. Ann. N Y Acad. Sci..

[5] Finkel T., Holbrook N.J. (2000). Oxidants, oxidative stress and the biology of ageing. Nature.

[6] Finkel T. (1998). Oxygen radicals and signaling. Curr. Opin. Cell Biol..

[7] Thannickal V.J., Fanburg B.L. (2000). Reactive oxygen species in cell signaling. Am. J. Physiol. Lung Cell Mol. Physiol..

[8] Kang D.H. (2002). Oxidative stress, DNA damage, and breast cancer. AACN Clin. Issues.

[9] Michiels C., Minet E., Mottet D., Raes M. (2002). Regulation of gene expression by oxygen: NF-kappaB and HIF-1, two extremes. Free Radic. Biol. Med..

[10] Nordberg J., Arner E.S. (2001). Reactive oxygen species, antioxidants, and the mammalian thioredoxin system. Free Radic. Biol. Med..

[11] Wasserman W.W., Fahl W.E. (1997). Functional antioxidant responsive elements. Proc. Natl. Acad. Sci. USA.

[12] Ishii T., Itoh K., Takahashi S., Sato H., Yanagawa T., Katoh Y., Bannai S., Yamamoto M. (2000). Transcription factor Nrf2 coordinately regulates a group of oxidative stress-inducible genes in macrophages. J. Biol. Chem..

[13] Katoh Y., Iida K., Kang M. I., Kobayashi A., Mizukami M., Tong K. I., McMahon M., Hayes J.D., Itoh K., Yamamoto M. (2005). Evolutionary conserved N-terminal domain of Nrf2 is essential for the Keap1-mediated degradation of the protein by proteasome. Arch. Biochem. Biophys..

[14] Lee J.M., Johnson J.A. (2004). An important role of Nrf2-ARE pathway in the cellular defense mechanism. J. Biochem. Mol. Biol..

[15] Rocha S. (2007). Gene regulation under low oxygen: holding your breath for transcription. Trends. Biochem. Sci..

[16] Pouyssegur J., Dayan F., Mazure N.M. (2006). Hypoxia signalling in cancer and approaches to enforce tumour regression. Nature.

[17] Keith B., Simon M.C. (2007). Hypoxia-inducible factors, stem cells, and cancer. Cell.

[18] Matsui M., Oshima M., Oshima H., Takaku K., Maruyama T., Yodoi J., Taketo M.M. (1996). Early embryonic lethality caused by targeted disruption of the mouse thioredoxin gene. Dev. Biol..

[19] Holmgren A. (1985). Thioredoxin. Annu. Rev. Biochem..

[20] Arner E.S., Holmgren A. (2000). Physiological functions of thioredoxin and thioredoxin reductase. Eur. J. Biochem..

[21] Gromer S., Urig S., Becker K. (2004). The thioredoxin system--from science to clinic. Med. Res. Rev..

[22] Nishinaka Y., Nakamura H., Masutani H., Yodoi J. (2001). Redox control of cellular function by thioredoxin; a new therapeutic direction in host defence. Arch. Immunol. Ther. Exp. (Warsz).

[23] Powis G., Montfort W.R. (2001). Properties and biological activities of thioredoxins. Annu. Rev. Biophys. Biomol. Struct..

[24] Nakamura H., Nakamura K., Yodoi J. (1997). Redox regulation of cellular activation. Annu. Rev. Immunol..

[25] Taniguchi Y., Taniguchi-Ueda Y., Mori K., Yodoi J. (1996). A novel promoter sequence is involved in the oxidative stress-induced expression of the adult T-cell leukemia-derived factor (ADF)/human thioredoxin (Trx) gene. Nucleic Acids Res..

[26] Spyrou G., Enmark E., Miranda-Vizuete A., Gustafsson J. (1997). Cloning and expression of a novel mammalian thioredoxin. J. Biol. Chem..

[27] Miranda-Vizuete A., Ljung J., Damdimopoulos A.E., Gustafsson J.A., Oko R., Pelto-Huikko M., Spyrou G. (2001). Characterization of Sptrx, a novel member of the thioredoxin family specifically expressed in human spermatozoa. J. Biol. Chem..

[28] Sun Q.A., Su D., Novoselov S.V., Carlson B.A., Hatfield D.L., Gladyshev V.N. (2005). Reaction mechanism and regulation of mammalian thioredoxin/glutathione reductase. Biochemistry.

[29] Mitsui A., Hirakawa T., Yodoi J. (1992). Reactive oxygen-reducing and protein-refolding activities of adult T cell leukemia-derived factor/human thioredoxin. Biochem. Biophys. Res. Commun..

[30] Wood Z.A., Schroder E., Robin Harris J., Poole L.B. (2003). Structure, mechanism and regulation of peroxiredoxins. Trends Biochem. Sci..

[31] Rhee S.G., Chae H.Z., Kim K. (2005). Peroxiredoxins: a historical overview and speculative preview of novel mechanisms and emerging concepts in cell signaling. Free Radic. Biol. Med..

[32] Immenschuh S., Baumgart-Vogt E. (2005). Peroxiredoxins, oxidative stress, and cell proliferation. Antioxid. Redox Signal..

[33] Chae H.Z., Kim H.J., Kang S.W., Rhee S.G. (1999). Characterization of three isoforms of mammalian peroxiredoxin that reduce peroxides in the presence of thioredoxin. Diabetes Res. Clin. Pract..

[34] Bertini R., Howard O.M., Dong H.F., Oppenheim J.J., Bizzarri C., Sergi R., Caselli G., Pagliei S., Romines B., Wilshire J.A., Mengozzi M., Nakamura H., Yodoi J., Pekkari K., Gurunath R., Holmgren A., Herzenberg L.A., Ghezzi P. (1999). Thioredoxin, a redox enzyme released in infection and inflammation, is a unique chemoattractant for neutrophils, monocytes, and T cells. J. Exp. Med..

[35] Kondo N., Nakamura H., Masutani H., Yodoi J. (2006). Redox regulation of human thioredoxin network. Antioxid. Redox. Signal..

[36] Hirota K., Murata M., Sachi Y., Nakamura H., Takeuchi J., Mori K., Yodoi J. (1999). Distinct roles of thioredoxin in the cytoplasm and in the nucleus. A two-step mechanism of redox regulation of transcription factor NF-kappaB. J. Biol. Chem..

[37] Demple B., Herman T., Chen D.S. (1991). Cloning and expression of APE, the cDNA encoding the major human apurinic endonuclease: definition of a family of DNA repair enzymes. Proc. Natl. Acad. Sci. USA.

[38] Robson C.N., Hickson I.D. (1991). Isolation of cDNA clones encoding a human apurinic/apyrimidinic endonuclease that corrects DNA repair and mutagenesis defects in E. coli xth (exonuclease III) mutants. Nucleic Acids Res..

[39] Robson C.N., Milne A.M., Pappin D.J., Hickson I.D. (1991). Isolation of cDNA clones encoding an enzyme from bovine cells that repairs oxidative DNA damage in vitro: homology with bacterial repair enzymes. Nucleic Acids Res..

[40] Hirota K., Matsui M., Iwata S., Nishiyama A., Mori K., Yodoi J. (1997). AP-1 transcriptional activity is regulated by a direct association between thioredoxin and Ref-1. Proc. Natl. Acad. Sci. USA.

[41] Wisdom R. (1999). AP-1: one switch for many signals. Exp. Cell. Res..

[42] Leppa S., Bohmann D. (1999). Diverse functions of JNK signaling and c-Jun in stress response and apoptosis. Oncogene.

[43] Angel P., Karin M. (1991). The role of Jun, Fos and the AP-1 complex in cell-proliferation and transformation. Biochim. Biophys. Acta.

[44] Abate C., Patel L., Rauscher F.J., Curran T. (1990). Redox regulation of fos and jun DNA-binding activity *in vitro*. Science.

[45] Ema M., Hirota K., Mimura J., Abe H., Yodoi J., Sogawa K., Poellinger L., Fujii-Kuriyama Y. (1999). Molecular mechanisms of transcription activation by HLF and HIF1alpha in response to hypoxia: their stabilization and redox signal-induced interaction with CBP/p300. EMBO J..

[46] Otaki M., Hatano M., Kobayashi K., Ogasawara T., Kuriyama T., Tokuhisa T. (2000). Cell cycle-dependent regulation of TIAP/m-survivin expression. Biochim. Biophys. Acta.

[47] Stehlik C., de Martin R., Kumabashiri I., Schmid J.A., Binder B.R., Lipp J. (1998). Nuclear factor (NF)-kappaB-regulated X-chromosome-linked iap gene expression protects endothelial cells from tumor necrosis factor alpha-induced apoptosis. J. Exp. Med..

[48] Wang C.Y., Mayo M.W., Korneluk R.G., Goeddel D.V., Baldwin A.S. (1998). NF-kappaB antiapoptosis: induction of TRAF1 and TRAF2 and c-IAP1 and c-IAP2 to suppress caspase-8 activation. Science.

[49] Matthews J.R., Wakasugi N., Virelizier J.L., Yodoi J., Hay R.T. (1992). Thioredoxin regulates the DNA binding activity of NF-kappa B by reduction of a disulphide bond involving cysteine 62. Nucleic Acids Res..

[50] Saitoh M., Nishitoh H., Fujii M., Takeda K., Tobiume K., Sawada Y., Kawabata M., Miyazono K., Ichijo H. (1998). Mammalian thioredoxin is a direct inhibitor of apoptosis signal-regulating kinase (ASK) 1. EMBO J..

[51] Wei S.J., Botero A., Hirota K., Bradbury C.M., Markovina S., Laszlo A., Spitz D.R., Goswami P.C., Yodoi J., Gius D. (2000). Thioredoxin nuclear translocation and interaction with redox factor-1 activates the activator protein-1 transcription factor in response to ionizing radiation. Cancer Res..

[52] Tanaka T., Nishiyama Y., Okada K., Hirota K., Matsui M., Yodoi J., Hiai H., Toyokuni S. (1997). Induction and nuclear translocation of thioredoxin by oxidative damage in the mouse kidney: independence of tubular necrosis and sulfhydryl depletion. Lab. Invest..

[53] Holmgren A. (1985). Thioredoxin. Annu. Rev. Biochem..

[54] Osborne S.A., Hawkes H.J., Baldwin B.L., Alexander K.A., Svingen T., Clarke F.M., Tonissen K.F. (2006). The tert-butylhydroquinone-mediated activation of the human thioredoxin gene reveals a novel promoter structure. Biochem. J..

[55] Kim Y.C., Masutani H., Yamaguchi Y., Itoh K., Yamamoto M., Yodoi J. (2001). Hemin-induced activation of the thioredoxin gene by Nrf2. A differential regulation of the antioxidant responsive element by a switch of its binding factors. J. Biol. Chem..

[56] Rundlof A.K., Carlsten M., Arner E.S. (2001). The core promoter of human thioredoxin reductase 1: cloning, transcriptional activity, and Oct-1, Sp1, and Sp3 binding reveal a housekeeping-type promoter for the AU-rich element-regulated gene. J. Biol. Chem..

[57] Rundlof A.K., Janard M., Miranda-Vizuete A., Arner E.S. (2004). Evidence for intriguingly complex transcription of human thioredoxin reductase 1. Free Radic. Biol. Med..

[58] Hintze K.J., Wald K.A., Zeng H., Jeffery E.H., Finley J.W. (2003). Thioredoxin reductase in human hepatoma cells is transcriptionally regulated by sulforaphane and other electrophiles *via* an antioxidant response element. J. Nutr..

[59] Takagi Y., Mitsui A., Nishiyama A., Nozaki K., Sono H., Gon Y., Hashimoto N., Yodoi J. (1999). Overexpression of thioredoxin in transgenic mice attenuates focal ischemic brain damage. Proc. Natl. Acad. Sci. USA.

[60] Ramana C.V., Boldogh I., Izumi T., Mitra S. (1998). Activation of apurinic/apyrimidinic endonuclease in human cells by reactive oxygen species and its correlation with their adaptive response to genotoxicity of free radicals. Proc. Natl. Acad. Sci. USA.

[61] Yao K.S., Xanthoudakis S., Curran T., O'Dwyer P.J. (1994). Activation of AP-1 and of a nuclear redox factor, Ref-1, in the response of HT29 colon cancer cells to hypoxia. Mol. Cell. Biol..

[62] Olinski R., Zastawny T., Budzbon J., Skokowski J., Zegarski W., Dizdaroglu M. (1992). DNA base modifications in chromatin of human cancerous tissues. FEBS Lett..

[63] Jaruga P., Zastawny T.H., Skokowski J., Dizdaroglu M., Olinski R. (1994). Oxidative DNA base damage and antioxidant enzyme activities in human lung cancer. FEBS Lett..

[64] Szatrowski T.P., Nathan C.F. (1991). Production of large amounts of hydrogen peroxide by human tumor cells. Cancer Res..

[65] Morriss G.M., New D.A. (1979). Effect of oxygen concentration on morphogenesis of cranial neural folds and neural crest in cultured rat embryos. J. Embryol. Exp. Morphol..

[66] Kallio P.J., Wilson W.J., O'Brien S., Makino Y., Poellinger L. (1999). Regulation of the hypoxia-inducible transcription factor 1alpha by the ubiquitin-proteasome pathway. J. Biol. Chem..

[67] Huang L.E., Gu J., Schau M., Bunn H.F. (1998). Regulation of hypoxia-inducible factor 1alpha is mediated by an O2-dependent degradation domain *via* the ubiquitin-proteasome pathway. Proc. Natl. Acad. Sci. USA.

[68] Salceda S., Caro J.  (1997). Hypoxia-inducible factor 1alpha (HIF-1alpha) protein is rapidly degraded by the ubiquitin-proteasome system under normoxic conditions. Its stabilization by hypoxia depends on redox-induced changes. J. Biol. Chem..

[69] Salceda S., Beck I., Srinivas V., Caro J. (1997). Complex role of protein phosphorylation in gene activation by hypoxia. Kidney Int..

[70] Li F., Sonveaux P., Rabbani Z.N., Liu S., Yan B., Huang Q., Vujaskovic Z., Dewhirst M.W., Li C.Y. (2007). Regulation of HIF-1alpha stability through S-nitrosylation. Mol. Cell..

[71] Chandel N.S., McClintock D.S., Feliciano C.E., Wood T.M., Melendez J.A., Rodriguez A.M., Schumacker P.T. (2000). Reactive oxygen species generated at mitochondrial complex III stabilize hypoxia-inducible factor-1alpha during hypoxia: a mechanism of O2 sensing. J. Biol. Chem..

[72] Mansfield K.D., Guzy R.D., Pan Y., Young R.M., Cash T.P., Schumacker P.T., Simon M.C. (2005). Mitochondrial dysfunction resulting from loss of cytochrome c impairs cellular oxygen sensing and hypoxic HIF-alpha activation. Cell Metab..

[73] Bell E.L., Klimova T.A., Eisenbart J., Moraes C.T., Murphy M.P., Budinger G.R., Chandel N.S. (2007). The Qo site of the mitochondrial complex III is required for the transduction of hypoxic signaling *via* reactive oxygen species production. J. Cell. Biol..

[74] Kietzmann T., Gorlach A. (2005). Reactive oxygen species in the control of hypoxia-inducible factor-mediated gene expression. Semin. Cell. Dev. Biol..

[75] Guzy R.D., Hoyos B., Robin E., Chen H., Liu L., Mansfield K.D., Simon M.C., Hammerling U., Schumacker P. T. (2005). Mitochondrial complex III is required for hypoxia-induced ROS production and cellular oxygen sensing. Cell. Metab..

[76] Wang G.L., Jiang B.H., Semenza G.L. (1995). Effect of altered redox states on expression and DNA-binding activity of hypoxia-inducible factor 1. Biochem. Biophys. Res. Commun..

[77] Liu Q., Berchner-Pfannschmidt U., Moller U., Brecht M., Wotzlaw C., Acker H., Jungermann K., Kietzmann T. (2004). A Fenton reaction at the endoplasmic reticulum is involved in the redox control of hypoxia-inducible gene expression. Proc. Natl. Acad. Sci. USA.

[78] Welsh S.J., Bellamy W.T., Briehl M.M., Powis G. (2002). The redox protein thioredoxin-1 (Trx-1) increases hypoxia-inducible factor 1alpha protein expression: Trx-1 overexpression results in increased vascular endothelial growth factor production and enhanced tumor angiogenesis. Cancer Res..

[79] Csiki I., Yanagisawa K., Haruki N., Nadaf S., Morrow J.D., Johnson D.H., Carbone D.P. (2006). Thioredoxin-1 modulates transcription of cyclooxygenase-2 *via* hypoxia-inducible factor-1alpha in non-small cell lung cancer. Cancer Res..

[80] Jones D.T., Pugh C.W., Wigfield S., Stevens M.F., Harris A.L. (2006). Novel thioredoxin inhibitors paradoxically increase hypoxia-inducible factor-alpha expression but decrease functional transcriptional activity, DNA binding, and degradation. Clin. Cancer Res..

[81] Berggren M., Gallegos A., Gasdaska J.R., Gasdaska P.Y., Warneke J., Powis G. (1996). Thioredoxin and thioredoxin reductase gene expression in human tumors and cell lines, and the effects of serum stimulation and hypoxia. Anticancer Res..

[82] Kim H.J., Chae H.Z., Kim Y.J., Kim Y.H., Hwangs T.S., Park E.M., Park Y.M. (2003). Preferential elevation of Prx I and Trx expression in lung cancer cells following hypoxia and in human lung cancer tissues. Cell. Biol. Toxicol..

[83] Hedley D., Pintilie M., Woo J., Nicklee T., Morrison A., Birle D., Fyles A., Milosevic M., Hill R. (2004). Up-regulation of the redox mediators thioredoxin and apurinic/apyrimidinic excision (APE)/Ref-1 in hypoxic microregions of invasive cervical carcinomas, mapped using multispectral, wide-field fluorescence image analysis. Am. J. Pathol..

[84] Folkman J. (1971). Tumor angiogenesis: therapeutic implications. N. Engl. J. Med..

[85] Han H., Bearss D.J., Browne L.W., Calaluce R., Nagle R.B., Von Hoff D.D. (2002). Identification of differentially expressed genes in pancreatic cancer cells using cDNA microarray. Cancer Res..

[86] Raffel J., Bhattacharyya A.K., Gallegos A., Cui H., Einspahr J.G., Alberts D.S., Powis G. (2003). Increased expression of thioredoxin-1 in human colorectal cancer is associated with decreased patient survival. J. Lab. Clin. Med..

[87] Choi J.H., Kim T.N., Kim S., Baek S.H., Kim J.H., Lee S.R., Kim J.R. (2002). Overexpression of mitochondrial thioredoxin reductase and peroxiredoxin III in hepatocellular carcinomas. Anticancer Res..

[88] Grogan T.M., Fenoglio-Prieser C., Zeheb R., Bellamy W., Frutiger Y., Vela E., Stemmerman G., Macdonald J., Richter L., Gallegos A., Powis G. (2000). Thioredoxin, a putative oncogene product, is overexpressed in gastric carcinoma and associated with increased proliferation and increased cell survival. Hum. Pathol..

[89] Cha M.K., Suh K.H., Kim I.H. (2009). Overexpression of peroxiredoxin I and thioredoxin1 in human breast carcinoma. J. Exp. Clin. Cancer. Res..

[90] Gallegos A., Gasdaska J.R., Taylor C.W., Paine-Murrieta G.D., Goodman D., Gasdaska P.Y., Berggren M., Briehl M.M., Powis G. (1996). Transfection with human thioredoxin increases cell proliferation and a dominant-negative mutant thioredoxin reverses the transformed phenotype of human breast cancer cells. Cancer Res..

[91] Lu J., Chew E.H., Holmgren A. (2007). Targeting thioredoxin reductase is a basis for cancer therapy by arsenic trioxide. Proc. Natl. Acad. Sci. U S A.

[92] Ceccarelli J., Delfino L., Zappia E., Castellani P., Borghi M., Ferrini S., Tosetti F., Rubartelli A. (2008). The redox state of the lung cancer microenvironment depends on the levels of thioredoxin expressed by tumor cells and affects tumor progression and response to prooxidants. Int. J. Cancer.

[93] Farina A.R., Tacconelli A., Cappabianca L., Masciulli M.P., Holmgren A., Beckett G.J., Gulino A., Mackay A.R. (2001). Thioredoxin alters the matrix metalloproteinase/tissue inhibitors of metalloproteinase balance and stimulates human SK-N-SH neuroblastoma cell invasion. Eur. J. Biochem..

[94] Lincoln D.T., Ali Emadi E.M., Tonissen K.F., Clarke F.M. (2003). The thioredoxin-thioredoxin reductase system: over-expression in human cancer. Anticancer Res..

[95] Chaiswing L., Bourdeau-Heller J.M., Zhong W., Oberley T.D. (2007). Characterization of redox state of two human prostate carcinoma cell lines with different degrees of aggressiveness. Free Radic. Biol. Med..

[96] Kim S.J., Miyoshi Y., Taguchi T., Tamaki Y., Nakamura H., Yodoi J., Kato K., Noguchi S. (2005). High thioredoxin expression is associated with resistance to docetaxel in primary breast cancer. Clin. Cancer Res..

[97] Iwao-Koizumi K., Matoba R., Ueno N., Kim S. J., Ando A., Miyoshi Y., Maeda E., Noguchi S., Kato K. (2005). Prediction of docetaxel response in human breast cancer by gene expression profiling. J. Clin. Oncol..

[98] Sasada T., Iwata S., Sato N., Kitaoka Y., Hirota K., Nakamura K., Nishiyama A., Taniguchi Y., Takabayashi A., Yodoi J. (1996). Redox control of resistance to cis-diamminedichloroplatinum (II) (CDDP): protective effect of human thioredoxin against CDDP-induced cytotoxicity. J. Clin. Invest..

[99] Marks P.A. (2006). Thioredoxin in cancer--role of histone deacetylase inhibitors. Semin. Cancer Biol..

[100] Yoshioka J., Schreiter E.R., Lee R.T. (2006). Role of thioredoxin in cell growth through interactions with signaling molecules. Antioxid. Redox. Signal..

[101] Butler L.M., Zhou X., Xu W.S., Scher H.I., Rifkind R.A., Marks P.A., Richon V.M. (2002). The histone deacetylase inhibitor SAHA arrests cancer cell growth, up-regulates thioredoxin-binding protein-2, and down-regulates thioredoxin. Proc. Natl. Acad. Sci. USA.

[102] Powis G., Kirkpatrick D.L. (2007). Thioredoxin signaling as a target for cancer therapy. Curr. Opin. Pharmacol..

[103] Arner E.S., Holmgren A. (2006). The thioredoxin system in cancer. Semin. Cancer Biol..

[104] Mukherjee A., Martin S.G. (2008). The thioredoxin system: a key target in tumour and endothelial cells. Br. J. Radiol..

[105] Tonissen K.F., Di Trapani G. (2009). Thioredoxin system inhibitors as mediators of apoptosis for cancer therapy. Mol. Nutr. Food Res..

[106] Douer D., Tallman M.S. (2005). Arsenic trioxide: new clinical experience with an old medication in hematologic malignancies. J. Clin. Oncol..

[107] Gromer S., Arscott L.D., Williams C.H.Jr., Schirmer R.H., Becker K. (1998). Human placenta thioredoxin reductase. Isolation of the selenoenzyme, steady state kinetics, and inhibition by therapeutic gold compounds. J. Biol. Chem..

[108] Hashemy S.I., Holmgren A. (2008). Regulation of the catalytic activity and structure of human thioredoxin 1 *via* oxidation and S-nitrosylation of cysteine residues. J. Biol. Chem..

[109] Fang J., Holmgren A. (2006). Inhibition of thioredoxin and thioredoxin reductase by 4-hydroxy-2-nonenal in vitro and in vivo. J. Am. Chem. Soc..

[110] Wang Y., Zhang H., Holmgren A., Tian W., Zhong L. (2008). Inhibitory effect of green tea extract and (-)-epigallocatechin-3-gallate on mammalian thioredoxin reductase and HeLa cell viability. Oncol. Rep..

[111] Du Y., Wu Y., Cao X., Cui W., Zhang H., Tian W., Ji M., Holmgren A., Zhong L. (2009). Inhibition of mammalian thioredoxin reductase by black tea and its constituents: implications for anticancer actions. Biochimie.

[112] Dewhirst M.W., Ong E.T., Braun R.D., Smith B., Klitzman B., Evans S.M., Wilson D. (1999). Quantification of longitudinal tissue pO2 gradients in window chamber tumours: impact on tumour hypoxia. Br. J. Cancer.

[113] Secomb T.W., Hsu R., Dewhirst M.W., Klitzman B., Gross J.F. (1993). Analysis of oxygen transport to tumor tissue by microvascular networks. Int. J. Radiat. Oncol. Biol. Phys..

[114] Dewhirst M.W., Kimura H., Rehmus S.W., Braun R.D., Papahadjopoulos D., Hong K., Secomb T.W. (1996). Microvascular studies on the origins of perfusion-limited hypoxia. Br. J. Cancer Suppl..

[115] Brown J.M. (1979). Evidence for acutely hypoxic cells in mouse tumours, and a possible mechanism of reoxygenation. Br. J. Radiol..

[116] Reinhold H.S., Blachiwiecz B., Blok A. (1977). Oxygenation and reoxygenation in 'sandwich' tumours. Bibl. Anat..

[117] Yamaura H., Matsuzawa T. (1979). Tumor regrowth after irradiation; an experimental approach. Int. J. Radiat. Biol. Relat. Stud. Phys. Chem. Med..

[118] Lanzen J., Braun R.D., Klitzman B., Brizel D., Secomb T.W., Dewhirst M.W. (2006). Direct demonstration of instabilities in oxygen concentrations within the extravascular compartment of an experimental tumor. Cancer Res..

[119] Nehmeh S.A., Lee N.Y., Schroder H., Squire O., Zanzonico P.B., Erdi Y.E., Greco C., Mageras G., Pham H.S., Larson S.M., Ling C.C., Humm J. L. (2008). Reproducibility of intratumor distribution of (18)F-fluoromisonidazole in head and neck cancer. Int. J. Radiat. Oncol. Biol. Phys..

[120] Yuan G., Nanduri J., Bhasker C.R., Semenza G.L., Prabhakar N.R. (2005). Ca2+/calmodulin kinase-dependent activation of hypoxia inducible factor 1 transcriptional activity in cells subjected to intermittent hypoxia. J. Biol. Chem..

[121] Semenza G.L., Prabhakar N.R. (2007). HIF-1-dependent respiratory, cardiovascular, and redox responses to chronic intermittent hypoxia. Antioxid. Redox Signal..

[122] Maulik N., Sato M., Price B.D., Das D.K. (1998). An essential role of NFkappaB in tyrosine kinase signaling of p38 MAP kinase regulation of myocardial adaptation to ischemia. FEBS Lett..

[123] Maulik N., Das D.K. (2002). Potentiation of angiogenic response by ischemic and hypoxic preconditioning of the heart. J. Cell. Mol. Med..

[124] Maulik N., Engelman R.M., Rousou J.A., Flack J.E., Deaton D., Das D.K. (1999). Ischemic preconditioning reduces apoptosis by upregulating anti-death gene Bcl-2. Circulation.

[125] Maulik N., Goswami S., Galang N., Das D.K. (1999). Differential regulation of Bcl-2, AP-1 and NF-kappaB on cardiomyocyte apoptosis during myocardial ischemic stress adaptation. FEBS Lett..

[126] Maulik N., Sasaki H., Addya S., Das D.K. (2000). Regulation of cardiomyocyte apoptosis by redox-sensitive transcription factors. FEBS Lett..

[127] Das D.K., Engelman R.M., Kimura Y. (1993). Molecular adaptation of cellular defences following preconditioning of the heart by repeated ischaemia. Cardiovasc. Res..

[128] Flack J.E., Kimura Y., Engelman R.M., Rousou J.A., Iyengar J., Jones R., Das D.K. (1991). Preconditioning the heart by repeated stunning improves myocardial salvage. Circulation.

[129] Li G.C., Vasquez J.A., Gallagher K.P., Lucchesi B.R. (1990). Myocardial protection with preconditioning. Circulation.

[130] Murry C.E., Jennings R.B., Reimer K.A. (1986). Preconditioning with ischemia: a delay of lethal cell injury in ischemic myocardium. Circulation.

[131] Yagi K., Liu C., Bando T., Yokomise H., Inui K., Hitomi S., Wada H. (1994). Inhibition of reperfusion injury by human thioredoxin (adult T-cell leukemia-derived factor) in canine lung transplantation. J. Thorac. Cardiovasc. Surg..

[132] Nakamura H., Vaage J., Valen G., Padilla C.A., Bjornstedt M., Holmgren A. (1998). Measurements of plasma glutaredoxin and thioredoxin in healthy volunteers and during open-heart surgery. Free Radic. Biol. Med..

[133] Kihlstrom M. (1990). Protection effect of endurance training against reoxygenation-induced injuries in rat heart. J. Appl. Physiol..

[134] Isowa N., Yoshimura T., Kosaka S., Liu M., Hitomi S., Yodoi J., Wada H. (2000). Human thioredoxin attenuates hypoxia-reoxygenation injury of murine endothelial cells in a thiol-free condition. J. Cell. Physiol..

[135] Turoczi T., Chang V.W., Engelman R.M., Maulik N., Ho Y.S., Das D.K. (2003). Thioredoxin redox signaling in the ischemic heart: an insight with transgenic mice overexpressing Trx1. J. Mol. Cell. Cardiol..

[136] Maulik N., Yoshida T., Engelman R.M., Deaton D., Flack J.E., Rousou J.A., Das D.K. (1998). Ischemic preconditioning attenuates apoptotic cell death associated with ischemia/reperfusion. Mol. Cell. Biochem..

[137] Gurusamy N., Malik G., Gorbunov N.V., Das D.K. (2007). Redox activation of Ref-1 potentiates cell survival following myocardial ischemia reperfusion injury. Free Radic. Biol. Med..

[138] Malik G., Gorbounov N., Das S., Gurusamy N., Otani H., Maulik N., Goswami S., Das D.K. (2006). Ischemic preconditioning triggers nuclear translocation of thioredoxin and its interaction with Ref-1 potentiating a survival signal through the PI-3-kinase-Akt pathway. Antioxid. Redox. Signal..

[139] Hawaleshka A., Jacobsohn E. (1998). Ischaemic preconditioning: mechanisms and potential clinical applications. Can. J. Anaesth..

[140] Zhu Y., Ohlemiller K.K., McMahan B.K., Gidday J.M. (2002). Mouse models of retinal ischemic tolerance. Invest. Ophthalmol. Vis. Sci..

[141] Zimmermann C., Ginis I., Furuya K., Klimanis D., Ruetzler C., Spatz M., Hallenbeck J.M. (2001). Lipopolysaccharide-induced ischemic tolerance is associated with increased levels of ceramide in brain and in plasma. Brain Res..

[142] Simon R.P. (1999). Hypoxia versus ischemia. Neurology.

[143] Moolman J.A., Genade S., Winterbach R., Lochner A. (1994). Preconditioning with hypoxia versus global ischemia in the isolated rat heart: effect on function and metabolism. Cardioscience.

[144] Gidday J.M., Fitzgibbons J.C., Shah A.R., Park T.S. (1994). Neuroprotection from ischemic brain injury by hypoxic preconditioning in the neonatal rat. Neurosci. Lett..

[145] Emerson M.R., Nelson S.R., Samson F.E., Pazdernik T.L. (1999). A global hypoxia preconditioning model: neuroprotection against seizure-induced specific gravity changes (edema) and brain damage in rats. Brain Res. Protoc..

[146] Gage A.T., Stanton P.K. (1996). Hypoxia triggers neuroprotective alterations in hippocampal gene expression *via* a heme-containing sensor. Brain Res..

[147] Barone F.C., White R.F., Spera P.A., Ellison J., Currie R.W., Wang X., Feuerstein G.Z. (1998). Ischemic preconditioning and brain tolerance: temporal histological and functional outcomes, protein synthesis requirement, and interleukin-1 receptor antagonist and early gene expression. Stroke.

[148] Wick A., Wick W., Waltenberger J., Weller M., Dichgans J., Schulz J.B. (2002). Neuroprotection by hypoxic preconditioning requires sequential activation of vascular endothelial growth factor receptor and Akt. J. Neurosci..

[149] Bernaudin M., Tang Y., Reilly M., Petit E., Sharp F.R. (2002). Brain genomic response following hypoxia and re-oxygenation in the neonatal rat. Identification of genes that might contribute to hypoxia-induced ischemic tolerance. J. Biol. Chem..

[150] Bernaudin M., Nedelec A.S., Divoux D., MacKenzie E.T., Petit E., Schumann-Bard P. (2002). Normobaric hypoxia induces tolerance to focal permanent cerebral ischemia in association with an increased expression of hypoxia-inducible factor-1 and its target genes, erythropoietin and VEGF, in the adult mouse brain. J. Cereb. Blood Flow. Metab..

